# Patients’ and Health Care Professionals’ Perspectives on Remote Patient Monitoring in Chronic Obstructive Pulmonary Disease Exacerbation Management: Initiating Cocreation

**DOI:** 10.2196/67666

**Published:** 2025-05-26

**Authors:** Atena Mahboubian, Marise J Kasteleyn, Enna Bašić, Niels H Chavannes, Jiska J Aardoom

**Affiliations:** 1 Department of Public Health and Primary Care, National eHealth Living Lab (NeLL) Leiden University Medical Center Leiden The Netherlands

**Keywords:** remote monitoring, chronic obstructive pulmonary disease, COPD, exacerbation management, eHealth, cocreation, coanalysis, experiences, expectations, self-management

## Abstract

**Background:**

Chronic obstructive pulmonary disease (COPD) exacerbations cause physiological and psychological distress, affecting overall health and quality of life. Early diagnosis of exacerbations is crucial for preserving lung function, preventing hospitalizations, and reducing health care costs. While remote patient monitoring (RPM) offers the potential for early exacerbation detection, challenges remain in recognizing symptoms in a timely manner. A noninvasive breath analysis device is under development to monitor patients with COPD and detect exacerbations before symptoms arise by measuring breath biomarkers through volatile organic compounds. This study encompassed the initial cocreation phase to align the use of the breath analysis device and corresponding care process with current COPD exacerbation management and user needs.

**Objective:**

We aimed to explore perspectives on COPD care processes, exacerbation management, and RPM in the Netherlands through 3 objectives: (1) identify stakeholders in COPD exacerbation care, (2) understand existing COPD care, and (3) explore stakeholder experiences and expectations regarding RPM in COPD care.

**Methods:**

Following the Center for eHealth Research and Disease Management Roadmap, 4 research activities were conducted between March 2024 and September 2024 for the initial cocreation phase: (1) desk research, (2) interviews, (3) project group meeting, and 4) coanalysis focus group. Desk research involved reviewing literature and COPD (exacerbation) care guidelines. Semistructured interviews (N=34) were conducted with 18 patients, 14 health care professionals (HCPs), 1 caregiver, and 1 hospital policy adviser. Topics included COPD diagnosis, exacerbation management, stakeholder roles in COPD care, and RPM experiences or expectations. The project group meeting between interviews and the focus group verified interim findings and guided the focus group content. In total, 6 patients participated in a coanalysis focus group to review interview quotes on exacerbations and RPM. The framework method was used to analyze the interviews and the focus group through abductive coding.

**Results:**

Seven key stakeholders were identified in COPD care, patients, pulmonologists, general practitioners, nurse practitioners, nurse specialists, physiotherapists, and informal caregivers. We observed a lack of uniformity in COPD care, exacerbation management, and information provision across HCPs. Patients reported struggling to recognize exacerbations. Although patients with experience in RPM reported positive experiences, they questioned the added value in early detection of exacerbations. Those without RPM experience were receptive to its use for symptom tracking but were concerned about reduced in-person care and overreliance on data. HCPs reported seeing value in RPM for between-visit monitoring and efficiently allocating resources but stressed the need for clear guidelines and noted barriers, such as language proficiency and technology usability.

**Conclusions:**

This study highlights the opportunities to improve COPD care and optimize exacerbation management with RPM. Future research should refine RPM processes, balance objective data with patient-reported symptoms, enhance communication among HCPs and with patients, provide clear exacerbation management guidelines, and ensure inclusivity.

## Introduction

### Background

Chronic obstructive pulmonary disease (COPD) is characterized by a gradual deterioration of pulmonary function and is the third leading cause of death worldwide [1]. The global financial burden is estimated to be US $4.5 trillion between 2020 and 2050 [2]. Symptoms like persistent cough, (exertional) dyspnea, wheezing, and sputum production, along with comorbidities, characterize COPD and impact patients’ quality of life (QoL) [3]. Patients can experience sudden worsening of symptoms, clinically identified as exacerbations increasing the risk of lung function deterioration [4,5].

Annual exacerbation rates range between 22% and 47%, with hospitalization rates varying from 5% to 25% [6,7]. Moreover, in the Netherlands, 15% of patients are readmitted within 30 days and 30% within a year [8]. Exacerbations strongly predict future exacerbations, significantly affecting patients’ physiological and psychological QoL [7,9]. Therefore, early exacerbation diagnosis and prevention are pivotal for enhancing QoL, preventing hospitalizations and readmissions, and reducing associated health care costs [9-13]. However, identifying exacerbations remains challenging, as it is difficult to distinguish them from typical COPD fluctuations [5,14]. Therefore, patients may struggle to recognize exacerbations and seek timely care, leading to acute worsening of symptoms and potential hospital visits or hospitalization [12,15].

Existing exacerbation management processes may facilitate early recognition. These processes include identifying personalized exacerbation indicators and determining tailored actions (ie, COPD action plan) or remote patient monitoring (RPM) to monitor parameters, such as oxygen saturation and physical activity [16-18]. This can help transition COPD care from reactive to proactive by increasing patient engagement and empowerment [19,20]. Some exacerbation management processes show promise in reducing delays in symptom recognition, decreasing exacerbation frequency, hospitalization duration, and readmissions [16,18,21]. Nevertheless, most processes fail to adequately predict exacerbations presymptomatically, possibly due to the lack of clear objective predictors and cut-off measures in combination with patient-reported outcomes [22-24]. In addition, existing processes still recognize usability issues largely due to the lack of trust, unclear processes, and labor-intensive tasks faced by end users. These challenges include frequent questionnaires, difficulty in expressing their feelings, the need to manage false alarms by monitoring staff, and lack of inclusivity by excluding more vulnerable patients with low health literacy [19,25-27]. These challenges may hinder adherence and widespread adoption, potentially limiting their benefits for a substantial portion of the COPD population, specifically the more vulnerable [23,24,28]. This highlights the disparity between the needs of exacerbation management processes, such as RPM, and existing processes [19,22,23].

A novel noninvasive RPM breath analysis device (Respiro BV) for COPD exacerbation management is currently under development to objectively predict exacerbations presymptomatically through specific exacerbation-indicative breath biomarkers (ie, volatile organic compounds) [29]. The concentration of these breath biomarkers will be measured through plasma emission spectroscopy to accurately identify the type and quantity of each biomarker present. They aim to ultimately create a unique breath print based on the presence and concentration of the biomarkers; this breath print will be analyzed by their proprietary algorithm to predict a possible exacerbation. However, the device is still under development with no product prototype available in clinical practice.

Research recommends involving relevant stakeholders and end users in early product development to improve user experience and promote successful adoption and to facilitate cocreation [24,30]. The definition of cocreation is fluid and is slightly open for interpretation; however, generally, it refers to the importance of involving diverse stakeholders in the process of defining complex problems, designing, and evaluating applicable solutions [31,32]. Cocreation enables the development of goods or services that are both valuable to prospective end users and aligned with their needs, as well as the specific context in which they are intended to be implemented [31]. The initial step in cocreation is often researching the context in which the product and process are to be implemented (ie, contextual inquiry) [33]. Therefore, in this phase of the breath analysis device development, a thorough understanding of COPD care and RPM processes is required by exploring prospective end users perspectives, expectations, and needs on integrating the use of a potential breath analysis device into COPD exacerbations management [23,24].

### Objectives

This study assists in the development of a novel RPM breath analysis device and corresponding care process by conducting a contextual inquiry. As such, it serves as the foundation for the continuation of the cocreation process for the breath analysis device [33]. This study aimed to thoroughly explore perspectives on COPD care processes, exacerbation management, and RPM in the Netherlands through following 3 objectives: (1) identify stakeholders involved in COPD exacerbation care, (2) understand existing COPD care with an explicit focus on exacerbations, and (3) explore stakeholder experiences and expectations regarding RPM in COPD care.

## Methods

### Study Design

#### Overview

This qualitative cocreation study encompasses the contextual inquiry, which is the initial cocreation phase according to the Center for eHealth Research and Disease Management (CeHRes) Roadmap [33]. The CeHRes Roadmap focuses on the development of eHealth technologies, emphasizing human characteristics, the socioeconomic and cultural environment, and the technology itself as interconnected and essential factors for successful development and adoption of eHealth solutions. Therefore, this roadmap is applicable in many different contexts and for different diseases or health-related issues [33]. The roadmap will guide the entire cocreation process of the breath analysis device, which is currently under development; therefore, it is crucial to understand the context in which the device will ultimately be implemented and identify successes and any existing difficulties. Therefore, this study focuses on the initial cocreation step, contextual inquiry. The next phase will be the value specification, which will focus on defining the needs, wishes, and values of prospective end users regarding the device and corresponding care process and translating them into product and process requirements. The design phase entails the development of prototypes, and the usability testing of these prototypes. Operationalization will examine the actual use of the device in the existing care process, and a summative evaluation be made for this use. The formative evaluation step is inherent to the iterative nature of cocreation and facilitates moments of evaluation [33].

**Figure 1:**
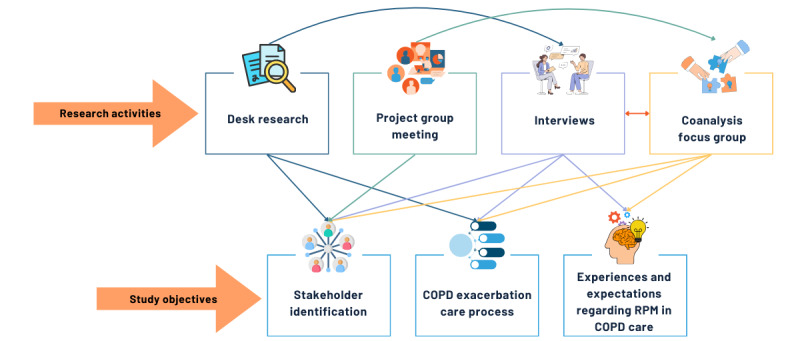
Overview of research activities in the contextual inquiry phase, and the corresponding contribution to the study objectives. The research activities are visualized in the boxes in the top row, with the arrows indicating the interdependencies between activities in terms of input or recruitment. The arrows between the research activities and research objectives show which activities have contributed to achieving the study’s objectives. COPD: chronic obstructive pulmonary disease; RPM: remote patient monitoring.

Within this study, as shown in Figure 1, the contextual inquiry phase encompassed 4 iterative research activities to achieve the research objectives as follows: (1) desk research, (2) in-depth interviews, (3) project group meeting, and (4) coanalysis focus group. Given the broad applicability of cocreation and the flexible interpretation of the CeHRes Roadmap, the research activities conducted in this study can be adapted for other chronic diseases to inform and guide cocreation processes [32,33].

#### Desk Research

The desk research focused on a search of gray literature (eg, COPD guidelines and recommendations) and scientific literature concerning COPD care, exacerbation management (ie, COPD action plans, personalized guides that helps patients recognize worsening symptoms early and provides specific steps for managing flare-ups to prevent further worsening), and existing RPM processes. This offered an initial overview of stakeholders and their relative importance based on a general understanding of COPD exacerbation management and RPM processes. In addition, during a COPD care conference, health care professional (HCPs) were asked to identify key stakeholders in COPD care. These insights were added to the stakeholder overview.

#### Interviews

Semistructured interviews were conducted with preliminarily identified stakeholders, mainly prospective end users, to obtain in-depth information concerning the perspectives and experiences of stakeholders regarding general COPD care, living with COPD, COPD exacerbation management, and RPM experiences or expectations, as well as to verify the preliminary stakeholder overview [34].

#### Project Group Meeting

One project group of key COPD stakeholders, including patients and HCPs, was formed to integrate cocreation throughout the research. The *project group* enabled stakeholders to participate in decision-making for subsequent research activities.

#### Coanalysis Focus Group

In a coanalysis focus group, we engaged patients to verify the preliminary stakeholder overview and information obtained from the interviews. Subsequently, participants were inquired to participate in the thematic analysis of interview quotes related to the following research topics, experiencing exacerbations, care received during exacerbations, and RPM expectations and experiences. The *coanalysis* *focus group* provided deeper insights into the interview data, helping researchers address their own biases by incorporating patient perspectives [35,36].

### Ethical Considerations

In the Netherlands, studies that do not fall under the scope of the Dutch Medical Research Involving Human Subjects Act (WMO) do not require a full ethical review by a recognized Medical-Ethical Review Committee. Instead, they are assessed by local non-WMO committees, which provide a declaration of no objection. Such a nonobjection declaration was also granted to this study by the non-WMO review board of Leiden University Medical Center (24-3015). This study is compliant with the General Data Protection Regulation and the Dutch Act on Implementation of the General Data Protection Regulation (Uitvoeringswet AVG). All participants provided written consent before participating in an interview or focus group. Patients and HCPs received €25 (US $28) and €100 (US $113) per hour interview, respectively.

### Recruitment and Participants

#### Overview

**Figure 2:**
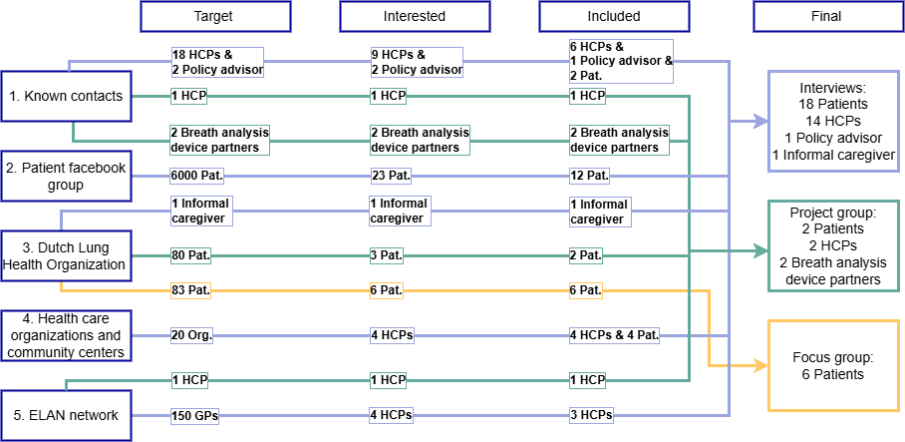
This overview provides a clear breakdown of the recruitment processes across various activities, contacts, and databases. The Target column represents the number of individuals targeted through each activity. The Interested column narrows this down to those who expressed interest following recruitment efforts. The Included column reflects the final number of individuals who were included based on the respective activity. Finally, the Final column visualizes the number of participants per research activity, as illustrated in Figure 1. ELAN: Extramural Leiden University Medical Center Academic Network; GP: general practitioner; HCP: health care professional; Pat: patient; Org: organization.

For the interviews, project meeting, and coanalysis focus group, purposeful sampling was used to strive for a diverse population based on characteristics such as age, sex, ethnicity, profession (for HCPs), and living situation (urban or rural) [37]. The target population comprised identified stakeholders, such as patients and HCPs involved in COPD exacerbation care. Patients were eligible if they had experienced or feared an exacerbation within the past 3 years, while HCPs needed to be actively working in COPD care. In addition, 2 non–end users (ie, hospital policy adviser for patient care and informal caregiver) were included. Sampling continued until saturation was reached, with no new themes emerging from the interviews, with the coanalysis focus group further confirming data saturation. In total, 5 main recruitment approaches were used. Figure 2 illustrates the various approaches and their contributions to the final number of participants in each research activity.

#### Interviews

All recruitment strategies were used. Familiar contacts were emailed or called accordingly. Health care organizations were emailed and called in cases of no response. The recruitment flyer and text were distributed in a Facebook group for pulmonology patients. The Extramural Leiden University Medical Center Academic Network for Primary Care Facilities database was targeted through a newsletter [38]. Finally, a patient’s partner from the coanalysis focus group was recruited as an informal caregiver (Figure 1). After prospective participants showed interest, they received a detailed information letter and informed consent form to formally consent to their participation. In total, 14 HCPs, 18 patients, 1 hospital policy adviser, and 1 informal caregiver were interviewed.

#### Project Group

The Lung Health Organization recruited patients from its own database of patients who were generally interested in contributing to COPD-related projects (recruitment strategy 3). In addition, 2 specialized COPD nurses from both primary and secondary care were recruited via strategy 1 and 5 (Figure 2). Finally, 2 Respiro B.V. partners participated as senior management and engineer. A total of 3 Leiden University Medical Center researchers (JJA, MJK, and AM) initiated the meeting.

#### Coanalysis Focus Group

Researcher AM contacted the Lung Health Organization in Utrecht (the Netherlands) to request assistance with recruiting participants for the focus group (recruitment strategy 3). Their existing contacts were approached to gauge interest, leading to a *coanalysis focus group* session with 6 participants, including the coordinator.

### Data Collection

#### Interviews

The interviews lasted approximately 60 minutes and were performed physically or through Microsoft Teams. Interview topics (Multimedia Appendices 1-3) concerned the COPD diagnosis process, experiences with COPD, exacerbations, exacerbation management, the roles, and responsibilities of different stakeholders in COPD exacerbation management, and their possible experiences or expectations with RPM processes. Participants filled out a demographic characteristic questionnaire. Patient interviews were mostly conducted in person, while HCP interviews were typically done digitally due to their time constraints. Interviews were conducted between April 2024 and September 2024. In total, 10 interviews were conducted with 2 researchers (AM and EB); the remaining interviews were conducted either by researcher AM or EB.

#### Project Meeting

The participants were asked to verify the preliminary stakeholder overview developed based on the desk research and interviews*.* Thereafter, the content of the coanalysis focus group was discussed with the participants to obtain their feedback. This led to significant changes in the coanalysis focus group content, particularly in the explanation and exercises designed to familiarize participants with thematic analysis. The patients involved in this project group were familiar with participating in research activities and interacting with HCPs or researchers; hence, they felt confident enough to raise their voice and provide constructive feedback.

#### Coanalysis Focus Group

A total of 3 researchers (MJK, JJA, and AM) led the coanalysis focus group, consisting of 3 phases. In phase 1, participants created a stakeholder overview of key stakeholders in COPD care and a word cloud reflecting words associated with their experiences or expectations of RPM. In phase 2, participants were introduced to coanalysis through a coding exercise, creating a word cloud from their COPD care experiences, grouping related terms, and assigning overarching themes (ie, brain mapping). These 2 phases were moderated by AM and were conducted with the entire group. During phase 3*,* participants read quotes, identified keywords (codes), and were encouraged by the moderators to group these codes under overarching themes, as practiced in phase 2. Simultaneously, the quotes prompted discussions about their own experiences with COPD care, exacerbation management, and RPM often confirming interview findings and introducing new insights. This phase was moderated by JJA and MJK. Afterward, participants completed a questionnaire gathering their sociodemographical information.

### Data Analyses

#### Overview

The interviews and coanalysis focus group data were audio recorded, transcribed verbatim, and pseudonymized. Furthermore, descriptive analyses (eg, mean and SD, number and percentage) of interview and focus group participants were performed in Microsoft Excel 365 to summarize participants’ sociodemographic characteristics.

The framework method was deemed most applicable to the explorative nature of this study and allowed for codes and themes to emerge inductively and deductively [39,40]. The first 2 interviews with patients and HCPs were individually coded by 2 independent researchers (AM and EB). Consequently, the codes were compared during consensus meetings to develop a comprehensive code scheme and to improve intercoder reliability [41]. Once consensus was reached, one researcher (AM) coded the remaining transcripts. A consensus meeting was conducted if any new codes emerged. The entire coding procedure was supervised by 2 researchers (JJA and MJK) and the final set of codes and subcodes was reviewed (Multimedia Appendix 4). If required, consensus meetings were scheduled with the senior researchers. The analysis of the interview data was performed using the Atlas.ti (Lumivero) software (version 23).

#### Coanalysis Focus Group

The input from phase 1 was summarized and resulted in a final overview of key stakeholders in COPD exacerbation management and an overview of their first associations with RPM in COPD care. The thematic coding exercise of phase 2 was summarized in a handwritten overview developed during the focus group. The new codes identified by the participants during phase 3 were added to the existing code scheme and applied to relevant quotes. The discussions between participants while reading the quotes were recorded, transcribed, summarized, and coded using the existing code scheme. Any new themes that emerged from the coanalysis focus group were used to further contextualize the results.

## Results

We aimed to address the following objectives: (1) identifying stakeholders in COPD care, (2) understanding current COPD care processes, and (3) assessing stakeholder experiences and expectations regarding RPM in COPD care. During this process, 2 overarching themes emerged, providing a comprehensive summary of the contextual inquiry phase as follows: (1) The regular COPD care process and (2) The exacerbation management process. The experiences and expectations related to RPM were interrelated to these 2 main themes.

### Population Characteristics

An overview of key stakeholders in COPD exacerbation care was determined based on all research activities. The stakeholders prominently involved were patients; partners or informal caregivers; primary, secondary, and rehabilitation care nurses; physiotherapists; pulmonologists; and general practitioners (GPs). Less frequently mentioned stakeholders, such as home care nurses, pharmacists, and breathing coaches were excluded from the interviews for clarity.

Table 1 presents a summary of the demographics of the patient interviews and coanalysis focus group. Patients’ ages varied between 48 and 86 years and their COPD Global Initiative for Obstructive Lung Disease classification ranged from stage 2 to 4. Most patients (23/24, 96%) had experienced at least one exacerbation in the last 12 months. Table 2 represents a summary of the HCP interviews. The HCPs’ ages varied from 33 to 60 years and their professions were pulmonologist, nurse specialist (NS; secondary care), rehabilitation nurse, GP, nurse practitioner (NP; primary care), and physiotherapist. Participants were mainly from the central and southern regions of the country.

**Table 1:** Patient characteristics of interview and coanalysis focus group participants (N=24)^a^.

Characteristics		Values
Age (y), mean (SD)		68.5 (8.9)
Male sex, n (%)		14 (58)
**Nationality, n (%)**		
	Dutch	21 (88)
	Dutch-Moluccan	1 (4)
	Dutch-Iranian	1 (4)
	Dutch-Turkish	1 (4)
COPD^b^ (y), mean (SD)^c^		10 (7)
**GOLD^d^ classification, n (%)**		
	2	3 (13)
	3	9 (37)
	4	7 (29)
	Unknown	5 (21)
**Education, n (%)**		
	Low (no education to high school)	10 (42)
	Intermediate (intermediate vocational education)	6 (25)
	High (BSc, MSc, or doctorate)	8 (33)
**Profession, n (%)**		
	Disabled for work	7 (29)
	Retired	14 (58)
	Volunteer	3 (13)
Experience with RPM^e^, n (%)		7 (29)

^a^In total, 18 patients and 1 informal caregiver participated in interviews and 6 patients participated in the coanalysis focus group. The characteristics of the informal caregiver was not added to the table. She was aged 78 years, Dutch, had experience with RPM because of her partner, had an intermediate education, and was disabled for work due to burn-out and her informal caregiver tasks.

^b^In total, 21 patients provided information on the number of years they had a diagnosis of COPD.

^c^COPD: chronic obstructive pulmonary disease.

**^d^**GOLD: Global Initiative For Chronic Obstructive Lung Disease.

^e^RPM: remote patient monitoring.

**Table 2:** Characteristics of interview participants, health care professionals, and the policy adviser (N=15).

Characteristics				Values
Age (y), mean (SD)				43.7 (9.5)
Male sex, n (%)				4 (27)
**Profession, n (%)**				
	Pulmonologist			6 (40)
	GP^a^ (primary care)			1 (7)
	**Nurse**			5 (33)
		NS^b^ (secondary care)	1 (7)	
		Rehabilitation nurse	1 (7)	
		NP^c^ (primary care)	3 (20)	
	Physiotherapist			2 (13)
	Policy adviser			1 (7)
Experience (y), mean (SD)				15.6 (10.3)
Experience in COPD^d^ (y), mean (SD)				10.3 (5.5)
Experience with RPM, n (%)^e^				6 (40)

^a^GP: general practitioner.

^b^NS: nurse specialist.

^c^NP: nurse practitioner.

^d^COPD: chronic obstructive pulmonary disease.

^e^RPM: remote patient monitoring.

### Regular COPD Care Process

#### Overview

According to the HCPs, the initial conversation for diagnosis is typically conducted by the GP or pulmonologist. Some pulmonologists indicated that due to time constraints, follow-up conversations are conducted by nurses. In total, 2 interviewed NPs indicated that they serve as the primary point of contact from the initial diagnosis onward, only consulting the GP when unfamiliar situations arise. Depending on patient’s medical status and COPD classification they are either under treatment in primary care or secondary care. The more complex cases are often referred to secondary care. Although many found it difficult to recall details, most patients noted that the information they received at diagnosis was limited, typically consisting of a brief explanation of COPD and instructions on how to use their inhalers:

The initial diagnosis made by a GP was “you have COPD, good luck living with it and there is nothing we can do.”

Patient 10

The patients interviewed reported feeling restricted in their daily lives and having to rely on others during routine activities like grocery shopping, cooking, cleaning, and sometimes even eating. Individuals living alone initially felt embarrassed to seek help but soon recognized its necessity, especially as their rehabilitation emphasized the importance of distributing their energy throughout the day, particularly during recovery from an exacerbation. HCPs observed that patients often struggle to grasp the progressive nature of COPD, leading to frustration as they adapt to life changes caused by their worsening condition:

When we’re out and I need to use the rest room, I need to take breaks frequently and end up occupying the rest room for a while. I often try to anticipate going to the rest room before leaving, which adds more stress to the situation.

Patient 1

Patients with stable symptoms typically attend consultations with their GP or pulmonologist once or twice a year. Despite regular interactions, ambiguity often exists among patients regarding their primary point of contact. When available, patients may also have biannual appointments with an NP or NS. Physician visits often last between 10 and 15 minutes, covering medication management and their health status. Some patients expressed doubts about the value of pulmonologist visits, citing limited time for in-depth discussions and a lack of understanding of their needs and preferences concerning medication and aids, such as oxygen. Some even felt their concerns and questions were overlooked or ignored, leading to dissatisfaction and the decision to change physicians. Others reported high satisfaction, valuing effective communication and prompt responses. Some could easily contact their HCPs whenever they felt anxious or experienced symptoms worsening. An illiterate interviewee received information verbally, which met his needs. However, despite repeated explanations, he had difficulty retaining detailed information about his condition but was proactive in seeking assistance when needed.

HCPs reported that they strive to deliver optimal and equitable care, but experience challenges in providing optimal care to patients facing information or language barriers. Although NPs and NSs often have more time to address disease-related needs and use tools like the Assessment of Burden of Chronic Conditions (which visualizes changes in patient-reported outcomes with color-coded balloons) and the “topic overview” placemat (which helps patients choose relevant topics for discussion), they still acknowledged and encountered barriers:

Low literacy and limited digital skills present a challenging situation where communication frequently lacks because it just does not get through. When a patient relies on a young child to translate, it further hinders effective interaction and creates personal resistance from my end.

NS

#### Experiences and Expectations of RPM During Regular COPD Care

Some patients, as well as the informal caregiver (8/25, 32%), were familiar with RPM for COPD, using mainly apps with daily, weekly, or monthly questionnaires that became more frequent when they indicated an increase in symptoms. While they valued the reassurance of ongoing monitoring, motivation was often external and depended on HCP’s recommendations or perceived benefits for HCPs. Despite a generally positive view, many questioned RPM’s specific benefits for their health or COPD management and struggled with vague or ambiguous questions and unhelpful automatic responses such as “refer to your COPD action plan” or “call your GP.” In addition, some patients noted that the repetitive nature of the questions could lead them to respond automatically, without consciously reflecting on their feelings and answers. A system that could objectively measure their status was therefore considered beneficial to them:

The question, “Do you feel normal?” can be answered with the option, “I feel normal” or “I feel better.” But better? That hasn’t been the case for a long time. I find it a bit suggestive, and the other possible answer implies something much worse, making it difficult questions to interpret and answer.

Patient 8

Patients unfamiliar with the concept of RPM had varying expectations. Some were enthusiastic about sharing their status with HCPs, believing it would enhance communication and disease management. Others worried that RPM might reduce human interaction and limit consultations to even lesser frequent intervals. In addition, patients questioned whether RPM systems could fully capture their well-being through stand-alone data. Some worried that HCPs unfamiliar with their medical history might misinterpret data and therefore miss crucial insights. Others were open to interacting with new HCPs but emphasized the need for evidence or trust that the system would positively impact their well-being. Generally, most patients did not foresee technical barriers to using RPM but stressed the need for comprehensive in-person training to aid adoption and empower them. A patient with a migration background, fluent in Dutch but with a history of addiction and lower education, reported difficulties with tasks like emailing and online banking. Despite this, he expressed a positive attitude toward using RPM for health monitoring and regular HCP assessments. He felt confident in his ability to use RPM effectively, provided he received thorough introductory training:

Yes, I find it challenging to send emails and manage online banking for instance, but an app like that [RPM] sounds great. It would allow me to keep track of my disease and health status, and I wouldn’t need to bother my nurse as much. They could simply guide me through the app on what to do. I think that would be really helpful.

Patient 18

Among the interviewees, RPM was solely implemented in secondary care; hence, from the HCPs only 1 NS, some pulmonologists, and the policy advisor (6/15, 40%) were familiar with these processes within COPD care. In addition, 2 pulmonologists from the same hospital had recently changed RPM systems due to technical system changes but had positive experiences with the previous system as it showed a reduction of exacerbation-related emergency room visits and hospitalization. The NS with RPM experience was optimistic about the future of RPM. He foresaw that through RPM time and resources could be allocated more efficiently, but also noted challenges related to digital and language barriers and some patient populations’ eligibility. HCPs generally indicated asking patients if they were interested in participating in RPM processes. However, they also reported that in many cases they would assess patients’ eligibility. According to the participant whose native language was Farsi, language should not pose a significant barrier in today’s society. He suggested that tools like Google Translate effectively eliminate communication obstacles, enabling individuals such as himself to participate more in such interventions:

You don’t notice much of it [effect of RPM] yet, but I think in the long term, as more people participate and we all become more familiar with this population, it [RPM] will help reduce the workload. We’ll see patients less frequently, maybe only once every year and a half. That’s where the real benefit should come from.

NS

HCPs without RPM experience (9/15, 60%) were generally open to its benefits, noting that regular insights into patients’ well-being could prompt timely interventions and reduce unnecessary visits. However, they expressed concerns about the frequency of use, the inclusion of patients who may benefit from RPM but are perceived as ineligible, and the distribution of roles across different HCPs and the patient. They also reported that having insight into patients’ medication use, especially changes that might not be communicated until a regular consultation, would be very beneficial. This information is considered crucial for understanding a patient’s disease trajectory:

There are situations where a patient repeatedly falls ill with varying symptoms, and after several rounds of treatment, both the doctor and the patient start to wonder, “Are we on the right path?” A home monitoring app could add significant value here, providing insights that the patient might struggle to recall.

Pulmonologist

### Exacerbation Care Process

#### Experiencing and Recognizing Exacerbations

Most patients reported being unaware of what an exacerbation was until they had experienced it one or more times, a finding that was confirmed by some HCPs. Many did not recall detailed information about exacerbations at diagnosis or during following consultations, aside from possibly receiving information in brochures. Some HCPs reported that they intentionally avoid discussing exacerbations during the initial consultation to prevent overwhelming patients. Instead, they may briefly mention that symptom fluctuations are inherent to COPD or choose to introduce the topic only after the patient has experienced an exacerbation. A patient during the coanalysis focus group coded quotes regarding this topic as “feeling ignorant” and “confused” due to a lack of understanding about what was happening to him and the implications of worsening symptoms:

I couldn’t come back; it just kept getting worse. I should have called for help sooner, but I was inexperienced and overwhelmed. I felt completely out of breath, struggling to stay afloat, and genuinely feared it was the end.

Patient 9

Overall, patients reported experiencing certain exacerbations as traumatic, characterized by sensations of suffocation, a state of disorientation, and feeling deadly ill. When asking patients how they would describe an exacerbation, the definitions and recognition varied greatly among patients. Some described an exacerbation as persisting and extreme illness-like symptoms, such as fever, extreme fatigue, and breathlessness, while others described them as short episodes of extreme breathlessness sometimes leading to a panic attack or having a “bad day.” Almost all patients admitted struggling at times with distinguishing a “normal” cold from an exacerbation. In addition, patient responses indicated that terms such as “exacerbations” and “pneumonia” are sometimes used interchangeably by HCPs. As one nurse clarified, while every case of pneumonia is an exacerbation, not every exacerbation is a case of pneumonia:

Interviewer: You mentioned earlier that you have experienced exacerbations.

Patient 1: Yes, yes. Quite a lot actually. I’ll have three good days, and then I feel worse. That’s essentially another exacerbation. Although, for many patients, it’s very difficult to recognize what an exacerbation really is.

HCPs define an exacerbation as a worsening of symptoms for at least 2 to 3 days, with increased coughing and green sputum. They noted that patients often have difficulty recognizing an exacerbation promptly, even with a COPD action plan designed to guide early symptom identification and management.

#### Acting Upon Exacerbations

Delays in seeking help and contacting HCPs remain a concern in COPD care, confirmed by both patients and HCPs. Most (26/39, 67%) of the participants, both patients and HCPs, were familiar with the COPD action plan or had heard of something similar. In the other cases, it was either not discussed or unknown. Nevertheless, some HCPs acknowledged that use of the plan is not integrated into their standard care process. Most patients who had undergone rehabilitation reported becoming acquainted with the COPD action plan during rehabilitation, where they received practical tips and theoretical information on effective exacerbation recognition and daily energy management. All patients who underwent rehabilitation were highly enthusiastic and felt that they gained significant insights into their condition:

It’s usually introduced to people who experience frequent exacerbations, and then the pulmonary nurse is involved to develop such a plan with them. Ideally, we would provide it to everyone, but we simply don’t have enough resources for that.

Pulmonologist

The use of the plan varies significantly, some patients follow it diligently, while others use it inconsistently or still face challenges in seeking timely help due to uncertainty about symptom severity or underestimation and trivialization of symptoms. In addition, friends, family, and physiotherapists often alert patients to worsening shortness of breath or other physical changes, prompting them to contact their HCP. Some patients in secondary care indicated that they often consult their GP first for advice and medication. While pulmonologists generally have no objections to patients contacting their GP, they prefer to be informed about any exacerbation treatment to monitor disease progression. The interviewed physiotherapists mentioned occasionally contacting patients’ GPs to inform, advise, or discuss the patients’ conditions, given their frequent contact with patients. However, this interaction is often perceived as one-way communication and generally lacks a well-established multidisciplinary collaboration:

I don’t use it [COPD action plan]. No, I don’t really need it. It’s intended for situations where someone is with me and I might have an exacerbation. So my friends know they can refer to the plan because that’s what it’s for. At least, that’s how I understood it. Or isn’t it?

Patient 2

In some cases, patients have a clear protocol for managing symptom worsening, such as contacting their HCP for medication advice or scheduling a physician visit. Some patients had established agreements with their physicians, allowing them to initiate treatment independently when suspecting an exacerbation. For instance, an illiterate patient used a designated phone number to reach an NS during specific hours, to address his worsening symptoms. One GP recommended patients to call if coughing persisted 2 days, despite the clear guidance, they still experienced delays in patients seeking medical help. Furthermore, many patients felt the general definition of an exacerbation did not match their symptoms, such as lack of persistent coughing, green sputum, or fever, confusing whether to contact their HCP:

I have a card with the phone numbers and names of the nurses. You can call them during specific hours if it seems like things are worsening, like a telephone consultation for COPD.

Patient 13

#### Experiences and Expectations of RPM During Exacerbations

Patients experienced that RPM often had a well-established process in place already for managing symptom worsening. Consequently, they questioned the app’s value in addressing exacerbations. They often did not consider using the app for worsening symptoms if they had already completed their weekly questionnaire, as it might have been sent on Monday, while symptoms began on Wednesday. Although some patients received advice from nurses monitoring the app varying from, “wait and see” to “increase medication usage,” or “visit your HCP for a check-up,” when reporting worsened symptoms. The majority (6/7, 86%) did not find it effective in preventing or identifying exacerbations earlier. One patient specifically mentioned not feeling more secure or empowered by the app. However, most patients viewed it positively, appreciating the continuous insights into their well-being over a longer timeframe, its visibility for HCPs, and the ability to easily contact HCPs when needed. Although they rarely used the chat functionality for urgent medical support:

You would naturally get a sense of whether things were improving or worsening. And if things weren’t going well, I would automatically receive a message from the nurses saying, “It seems like you’re not doing well, maybe you take more medication” or “Be cautious and if it worsens contact your GP.” Overall, I was quite pleased with it.

Patient 3

They rely on the information I provide. And that time the exacerbation happened on Wednesday, not on a Monday or Sunday. In such cases, I don’t think about it [name of application]. I don’t consider calling my pulmonologist, I call my GP first. I’m not saying they wouldn’t have helped if I had called, but the app wouldn’t have been useful in that situation.

Patient 9

The interviewed patients unfamiliar with RPM processes (17/24, 70%) had varied perspectives and expectations regarding its use in exacerbation management. Some felt it could enhance their understanding of COPD and enable earlier detection of exacerbations. These patients, who often already monitored their saturation, blood pressure, or heart rate, were open to using an objective measurement system for predicting exacerbations. Conversely, others expressed skepticism, emphasizing the importance of physically observing patients and evaluating their overall presentation, even if conducted during video consultation. Furthermore, most patients felt it would be more convenient to interact with HCPs familiar with their health status to avoid repetitive discussions, reduce data misinterpretation, and foster trust:

It would be great if there would be something that could detect it [exacerbation] before I do. It would feel like having a buddy that helps keep an eye on me.

Patient 5

Last time I mentioned over the phone that I wasn’t feeling well, but she said, ‘the data shows you are fine,’ and, in those situations, I shut down, because I think to myself, I’m not a machine. No, I can’t say I have much confidence in home monitoring.

Patient 10

Most HCPs (14/15, 93%), regardless of their experience with RPM, believed that RPM could aid in the earlier identification of exacerbations, with a system incorporating both objective and subjective measurements being the most beneficial. HCPs generally preferred patients to proactively notify them through the RPM system. HCPs should intervene only if the system indicates concerning outcomes without the patient initiating contact. One physiotherapist suggested that HCPs should be responsible for monitoring the data and contacting patients, as patients might not be skilled to interpret medical data:

I think a questionnaire and some form of objective measurements could add even more value, where the breath analysis device could anticipate an exacerbation for instance. But it would need to be combined with the symptoms a person is experiencing.

Pulmonologist

Overall, most of the participants (34/40, 85%) were receptive toward RPM and anticipated benefits in early exacerbation detection. However, various participants noted that successful implementation requires clear guidance, logistics, defined roles, and responsibilities between different HCPs and patients, and careful consideration of RPM’s content and frequency of use. In addition, maintaining a human element in COPD exacerbation management is crucial, as exacerbation presentations can strongly vary among patients:

Clear agreements need to be made about responsibilities, and if I’m not the primary caregiver, I don’t think I am the right person to handle it. Other arrangements can be made, but they need to be clear at the regional level. Otherwise, it becomes unmanageable for the pulmonologist, especially with agreements involving multiple general practitioners. It’s best to coordinate such arrangements regionally to avoid overwhelming the hospital.

GP

## Discussion

### Principal Findings

This study encompassed the initial stage of cocreation, a contextual inquiry, to support the development of a novel RPM device and corresponding care process for COPD exacerbation management. We identified 7 main stakeholders as prominently involved in COPD care: patients, pulmonologist, GP, NP, NS, physiotherapists, and informal caregivers. Our findings revealed a lack of national uniformity in COPD care, exacerbation management, and RPM use, as well as some concerns regarding the use and added value of RPM; we also confirmed that exacerbation recognition remains challenging.

### Comparison to Previous Work

State-of-the-art literature was compared to our findings based on themes identified in the results. The implications of patients’ daily lives found in our study were in line with existing literature where patients often address their forced reliance on others due to their physical impairments and their inability to conduct simple tasks and domestic chores [42]. Furthermore, COPD is often linked to mental health challenges as patients cope with accepting their diagnosis, declining health, daily adjustments, and exacerbations [43]. These challenges may be reinforced by the lack of information provided to participants regarding exacerbations at diagnosis and throughout the COPD care process, which is a known issue in COPD care [25,44]. Hayes et al [45] conducted a questionnaire among UK patients to assess their perspectives on COPD care, where patients reported either missing information about their COPD diagnosis or during discharge, or not understanding the information provided. In addition, a review exploring patients’ needs in advanced stages of COPD concluded that information about the implications of the disease is often insufficient [46]. These findings suggest a lack of information provision experienced by patients throughout various stages of their disease [42,44-46]. Research shows that open communication and information provision strengthen the patient-HCP relationship [46]. This aligns with our findings that patients sometimes feel overlooked by their pulmonologist when their questions are ignored or when there is a lack of transparency about their health status. Our study, along with previous research, confirms that patients with COPD are eager to learn more about their condition [47]. However, the British Lung Foundation reported that approximately 75% of respondents did not receive clear support to manage their care and missed an agreed written plan on managing their COPD [48]. This emphasizes the importance of improving the guidance and involvement of patients with COPD throughout their care. Our study’s findings strengthen the advice of frameworks, such as patient-centered care where it is deemed essential to actively engage patients in their care process to improve health care quality and safety [49]. This prerequisite in patient-centered care can be translated to the development and implementation of sustainable and valuable RPM processes in COPD exacerbation management, by recognizing patients as collaborative experts and responding to their needs and values. In addition, HCPs noted that language barriers often diminish the quality of care and information provision. Borge et al [50] highlight this gap, noting that there is a disparity between the needs of patients with COPD with low health literacy and the ability of HCPs to address those needs. This lack of understanding of patients’ needs may reflect situations where language barriers impact the quality of care provided and received, suggesting that training and enhanced communication are necessary to bridge this gap [50].

The gaps and inequities in health care appear to widen with technical processes like RPM [51]. HCPs who participated in this study acknowledged that they gauge patients’ suitability for processes, such as RPM, a finding consistent with existing literature [52]. Studies highlight that HCPs often hesitate to offer RPM to their most vulnerable patients, limiting participation among those who could benefit the most [15,26,53]. These factors may unintentionally widen the gap within the Dutch COPD population by favoring patients who are more engaged with their disease or more willing to participate in such processes [53]. Cocreation frameworks, such as the CeHRes and PROCEDURES emphasize the importance of collaborating with patients continuously throughout the development cycle [32,33]. Although they recognize the need of focusing on health literacy and digital or language barriers, they often lack explicit guidance on how and when to consider and involve more vulnerable populations. Integrating theories in which inequality barriers are considered, such as the Enhanced Chronic Care model could facilitate in strengthening cocreation processes by actively involving and advocating on behalf of vulnerable populations [54].

Despite concerns anticipated inequities in RPM and the skepticism in the literature regarding the effectiveness of other RPM devices, such as at-home spirometers or wearables, the HCPs interviewed in this study remained optimistic about RPM’s future [17,52,55]. However, they emphasized that its success depends on facilitating conditions, such as clear agreements regarding implementation, communication, and responsibility. This aligns with the unified theory of acceptance and use of technology (UTAUT) framework, in which one of the concepts considered as an important factor for influencing behavioral change is “facilitating conditions” [56].

Most patients (6/7, 86%) in this study who used RPM questioned its added value for exacerbation management, early exacerbation detection, and overall well-being. These patients were often knowledgeable about their condition and had already established clear communication with their HCPs regarding exacerbation management. According to the literature, this might be a reason that patients perceive RPM processes as redundant or cumbersome [25]. The absence of perceived added value aligns with findings from a study assessing the app used by some of our participants, which found no significant reduction in exacerbation-related hospitalizations [22]. Nevertheless, the review by Nagase et al [20] highlights that RPM can lead to better insights into their condition, disease control, reduced anxiety, and a decreased burden on family members. These benefits were not frequently mentioned by participants in this study, although they expressed feeling more secure due to easier communication with their HCPs and the benefit of continuous insights into their well-being. However, most patients did not experience or believe that the app they used contributed to earlier recognition or action in case of an exacerbation. Conversely, patients without RPM experience believed that RPM could aid in early recognition of exacerbations, aligning with a qualitative study in which patients felt it could enhance their awareness of symptom deterioration [25]. This highlights a discrepancy between the perceived and experienced added value of RPM in COPD care and exacerbation management. The UTAUT emphasizes the role of performance expectancy, which has a direct influence on willingness to use and behavioral change. In our study this is reflected by patients’ expectations of RPM benefits despite lack of prior experience with RPM. In contrast, experienced patients, as reflected in this study, expressed skepticism about its added value in exacerbation management. According to the UTAUT, experience is a key moderating variable in behavioral change [56]. This underscores the importance of considering the perceived usefulness and lived experience of an innovation in the early stages of development to anticipate on the desired behavioral change in the implementation phase. The updated CeHRes 2.0 Roadmap highlights the importance of behavior change techniques during cocreation, which will be taken into account in the subsequent phases of the cocreation process [57].

There is no standardized care process for managing exacerbations as it largely depends on the agreements between patients and HCPs as well as patients’ ability to recognize when action is required, aligning with our findings [58]. Many patients continue to struggle with distinguishing between disease-related fluctuations, common colds, and exacerbation symptoms [5,59]. A widely known tool for early exacerbation recognition is the COPD action plan, aiding patients in identifying deteriorating symptoms early and providing personalized guidance, such as adjusting medication or contacting HCPs [18,28]. Most interviewed patients were familiar with this plan, although HCPs acknowledged that they do not introduce this to every patient. However, some patients familiar with the plan, continued struggling with recognizing exacerbation-related symptoms. Schrijver et al [19] also reported other barriers, such as disliking daily symptom monitoring and the complexity of symptom diaries and action plans. Participants in this study expressed a willingness to monitor daily symptoms through RPM if they believed it would enhance their COPD care or overall health status. Literature offers no clear consensus on the efficacy of RPM in preventing exacerbations. Some studies suggest that RPM aids in early exacerbation detection, reduces exacerbation frequency, and lowers hospitalization rates, while others find little significant evidence [16,60,61]. Studies demonstrating beneficial outcomes typically focus on measuring vital parameters. They may also incorporate questionnaires like the Clinical COPD Questionnaire and assess physical activity through step counting, which could account for differences in experiences and study results [16,21,60].

Previous studies emphasize the positive complementary effect of measuring objective data alongside patients’ subjective experiences [23,52]. A wide variety of RPM devices and processes exist, yet we note the variety in perceived and experienced added value. This points to the need to refine existing RPM devices and processes to accommodate efficient, effective, and inclusive COPD exacerbation management for the individual patient while taking behavior change techniques into account. This is in line with the recently published PANACEA framework, which emphasizes the importance of the degree to which RPM processes meet needs in COPD care by assessing performance, disease management, costs, patient experience, clinical experience, research experience, and access. This framework strengthens the need of this current cocreation study and continuation of cocreating the RPM device and care processes by involving the important stakeholder’s experiences and ultimately facilitating successful uptake in clinical care [62].

### Strengths and Limitations

An important strength of this study is its uniqueness in which this study has performed the initial cocreation phase, guided by the CeHRes Roadmap, with such detail within this population. We have systematically performed various iterative research activities that strengthened the cocreative nature of this study and the final findings. In line with this, the analyses of the interviews were partly conduced in coanalysis with patients with COPD, which, to our knowledge, is the first study to have performed coanalysis with this population. The coanalysis allowed for the involvement of prospective end users throughout all the aspects of this first cocreation phase, and to move beyond the researchers’ perspective during analyses [36]. Furthermore, studies often focus solely on either patients or HCPs, neglecting to address both perspectives in COPD care, exacerbation management, and RPM processes, or fail to include caregivers and other relevant stakeholders hampering the comprehensiveness of cocreation in this respective field [30,63]. This study addressed this limitation by incorporating a variety of stakeholders and perspectives. Finally, cocreation processes frequently involve knowledgeable patients who have adequate technical or language proficiency, potentially increasing health disparities [64]. In contrast, this study involved individuals with diverse migration backgrounds, educational levels, and digital and literacy proficiencies from the outset of the cocreation process. This approach established a strong foundation for future cocreation phases and ensured inclusivity from the start, aiding to develop a novel RPM device and corresponding care process to accommodate a broad range of end users’ needs and requirements.

One of the limitations was that patients unfamiliar with RPM might have found it challenging to envision the processes and articulate their expectations, particularly those with language barriers or lower educational levels. However, involving individuals who have no prior knowledge or experience with RPM to understand their perceptions of its purpose and potential relevance to their care process can also be perceived as strength. In such cases, researchers attempted to explain RPM using an example of an existing process, without giving too many details to avoid biasing responses. Nevertheless, it is possible that patients’ responses about their ideal RPM device and process were biased, as their creativity may have been hampered after hearing the researcher’s explanation. This could limit the extent to which their answers reflected original, imaginative thinking and restricted out-of-the-box ideas. Furthermore, their perceived expectations may have been skewed by the way potential RPM processes were presented, leading to the possible idealization of RPM’s added value to their COPD care and overall well-being. Furthermore, most participants were from the midsouth of the Netherlands, which may limit the generalizability of the findings to the northern regions, as differences were observed even between adjacent areas. Given the greater remoteness and longer distances to hospitals in the northern Netherlands, the perceived and experienced added value of RPM processes may differ significantly from the findings of stakeholders in the central and southern regions of the country.

### Implications

This study provides a solid foundation for the subsequent cocreation steps (value specification, design phase, operationalization, and evaluation) in developing a novel RPM device and corresponding care process for COPD exacerbation management. As this device remains under development, the study supports the identification and extrapolation of user needs, preferences, and values. These insights serve as inputs for subsequent cocreation phases, guiding the translation of user values and needs into technical and user requirements during the value specification and design phases. This approach aims to guide the development of a prototype for a novel RPM device and an accompanying care process blueprint, such as the breath analysis device. In addition, the methodology of this study can serve as a guide for other cocreation efforts, whether in similar or different populations or conditions. The findings also offer several opportunities to enhance clinical practice in COPD exacerbation management and RPM processes. Future research should focus on further developing and improving RPM devices and especially corresponding processes. This involves striking the right balance between collecting objective measurements and subjective patient-reported symptoms through RPM, improving communication among HCPs and between HCPs and patients in both primary and secondary care, providing clear information and transparency on COPD, medication usage, health aids, exacerbation management, and improving inclusivity. It also involves refining how HCPs evaluate patients’ eligibility for exacerbation management processes, such as RPM, to ensure that all patients can benefit from new interventions rather than only those deemed sufficiently skilled.

### Conclusions

This paper provides a detailed overview of the context in which a novel RPM device and its corresponding care process may be implemented. It offers insights into key stakeholders, regular COPD care processes, the variation in exacerbation management, information provision, and experiences and expectations regarding RPM processes. Improving the information and transparency provided to patients about COPD and exacerbations is essential for optimizing COPD care. The overall willingness to use RPM presents opportunities to enhance COPD exacerbation management. However, this study highlights the need to identify and target appropriate patients for RPM, particularly those often perceived as ineligible due to technical or language barriers. In addition, the perceived value of RPM must be demonstrated to patients and HCPs to facilitate acceptance and uptake. The findings highlight opportunities to refine COPD exacerbation management and RPM processes by improving devices, balancing objective data with patient-reported symptoms, strengthening communication between patients and HCPs, and ensuring inclusivity by involving key stakeholders throughout the cocreation process starting from the initial phase.

## Appendix

Multimedia Appendix 1 Interview guide for health care professionals—contextual inquiry.

Multimedia Appendix 2 Interview guide for patients with remote patient monitoring experience—contextual inquiry.

Multimedia Appendix 3 Interview guide for patients without remote patient monitoring experience—contextual inquiry.

Multimedia Appendix 4 Themes and codes overview—contextual inquiry.

## Abbreviations

CeHRes: Center for eHealth Research and Disease Management
COPD: chronic obstructive pulmonary disease
GP: general practitioner
HCP: health care professional
NP: nurse practitioner
NS: nurse specialist
QoL: quality of life
RPM: remote patient monitoring
UTAUT: unified theory of acceptance and use of technology
WMO: Dutch Medical Research Involving Human Subjects Act

## Data Availability

The datasets generated or analyzed during this study are not publicly available due to the fact that the participants did not consent to share the data with people and parties other than the researchers but are available from the corresponding author on reasonable request, 5 years after publication. The prerequisite is a data transfer agreement, approved by the legal department of the requesting researcher and the legal department of Leiden University Medical Center.
