# Supplementary material for: Patients’ and Health Care Professionals’ Perspectives on Remote Patient Monitoring in Chronic Obstructive Pulmonary Disease Exacerbation Management: Initiating Cocreation
Source: J Med Internet Res. 2025 May 26;27:e67666. doi: 10.2196/67666 (PMC12149775; doi:10.2196/67666)
Supplement: Multimedia Appendix 1 [file jmir_v27i1e67666_app1.docx]

### Appendix 1: Semi-structured interview guide for healthcare professionals

1. **Introduction (5 min)**
   1. Welcome
   2. Introducing the study
   3. Consent forms
   4. Filling out the socio-demographic questionnaire
2. **Personal introduction (2 min)**
   1. Interviewer introduction
   2. Interviewee introduction
3. **General introduction to COPD (3 min)**
   1. From your point of view as a professional, how would you describe life with COPD?
   2. What are the different types of patient populations within the COPD target group?
      1. *Different personal and societal patient characteristics, differences in disease severity*
4. **Stakeholders involved in COPD and home monitoring care (7 min)**
   1. How would you describe your role in the COPD care process?
   2. Do you have experience with remote home monitoring of COPD or other diseases?
      1. **If yes for COPD**, what kind of COPD home monitoring process do you have experience with?
         1. What is your role in home monitoring?
      2. **Depending on whether or not you have experience**, what are your experiences/ideas and opinions about remote home monitoring in general and specifically for COPD? *Prompt:* *(positive/negative)*
         1. **If not experienced**, how would you envision your role in remote home monitoring?
   3. Which people, both healthcare professionals and non-healthcare professionals, do you think are important in remote home monitoring?
      1. How would you describe the roles and responsibilities of these individuals?
      2. What role does the patient play in this process?
         1. *Prompt: Passive, active*
   4. **Applicable if experience with remote home monitoring**, Would you like to change the division of roles and responsibilities of the people involved?
      1. What and why?
5. **The care process prior to an exacerbation (12 min)**
   1. From your perspective as a healthcare provider, how do you describe the COPD care for a patient prior to an exacerbation (i.e. if there is no suspicion or indication of an increase in symptoms yet)?
      1. *Prompt: Different clinical steps that can be taken/healthcare providers involved/when is this healthcare provider specifically involved*
      2. **If applicable**: what does the home monitoring process look like in this phase?
      3. What is the division of roles between primary and secondary care?
   2. What information is shared with the patient about COPD and the its care?
      1. *Prompt: And COPD exacerbation care?*
      2. How is the patient prepared for the possible recognition of an exacerbation?
         1. *Prompt: What information is shared/what actions need to be taken?*
      3. What steps does the patient go through if he/she experiences an increase in symptoms or thinks/expects to have an exacerbation?
      4. Are there any steps, measures or actions that are discussed with the patient to minimize symptoms and to reduce/prevent the risk of an exacerbation and hospitalization? If so, which ones?
         1. *Prompt: Who can be approached and when?*
      5. **In the case of remote home monitoring**, what does the process look like if a patient is expected to exacerbate based on the measured data/available information?
         1. *Prompt: If there is no home monitoring, what does this process look like? Might be a repetition of 5bii.*
      6. How do you think the patients experience the use and process of remote home monitoring in the phase prior to an exacerbation? (*prompts: reassuring, insecure, control?*
6. **The care process during and after an exacerbation (10 min)**

***During***

- 1. What does the care process look like once a patient has been diagnosed with an exacerbation?
     1. Which healthcare providers are involved in this phase from the diagnosis of an exacerbation, and when?
        1. *Prompt: Primary and secondary care*
  2. What information is shared with the patient in this phase about the care and any remote home monitoring after an exacerbation or after possible discharge?
     1. In your opinion, is this information sufficient for the patient to be confident at home in the (remote home monitoring) care process?

***After***

- 1. What does the care process look like after an exacerbation or after a discharge?
     1. What role does remote home monitoring play in this? Or do you expect that home monitoring can play a role in this?
     2. *Prompt: What does the care process look like if there is no remote home monitoring? Might repetition of 6C*
        1. *Prompt: Contact with patient/preventive actions to prevent subsequent exacerbation/provision of information*
  2. Which people are actively involved in this phase and, if applicable, in the remote home monitoring process?
     1. What are the roles and responsibilities?
     2. How is the contact with the patient?
     3. **If you have no experience with home monitoring**: *which people do you think would have a prominent role in remote home monitoring in the phase after an exacerbation? Might be repetition of 4c*

1. **Care process remote home monitoring (15 min)**
   1. ***If it relates to the healthcare provider:*** If you compare the group of patients without remote home monitoring with the patients who do use it, what is the most striking difference in the period before and after an exacerbation?
      1. *Prompt: Patient experiences*
      2. *Prompt: Caregiver experiences*
      3. *Prompt: Processes*
   2. Would remote home monitoring be suitable for the entire COPD population, including patients with comorbidities?
      1. Which patient populations should we specifically take into consideration when introducing remote home monitoring?
         1. *Prompt: Which subgroups?*
         2. *Prompt: And why?*
      2. In your opinion, how could we get the vulnerable (read, low-literate, low-economic status) population on board and benefit from remote home monitoring?
   3. Which comorbidities are relevant for the COPD patient population?
      1. To what extent do you experience that certain comorbidities have an impact on overall COPD care and the roll-out of remote home monitoring?
   4. What is an important aspect for you to successfully apply remote home monitoring in COPD care and why?
      1. What would you need/what did you need to confidentially apply remote home monitoring in practice?
   5. What do you think the ideal form of remote home monitoring looks like?
2. **Closing (2-5 min)**
   1. Are there any other points you would like to share about the COPD care process and remote home monitoring?
   2. Are we allowed to be approached you for follow-up research?
