# Supplementary material for: Patients’ and Health Care Professionals’ Perspectives on Remote Patient Monitoring in Chronic Obstructive Pulmonary Disease Exacerbation Management: Initiating Cocreation
Source: J Med Internet Res. 2025 May 26;27:e67666. doi: 10.2196/67666 (PMC12149775; doi:10.2196/67666)
Supplement: Multimedia Appendix 2 [file jmir_v27i1e67666_app2.docx]

# Appendix 2: Semi-structured interview guides for patients with RPM experience (approximately 60 minutes)

1. **Introduction (5 min)**
   1. Welcome
   2. Introduction to research and interview
   3. Consent forms
   4. Fill in the socio-demographic questionnaire
2. **Personal introduction (3 min)**
   1. Interviewer introduction
   2. Interviewee introduction
      1. Can you tell us a bit about yourself?
3. **General introduction to COPD (5 min)**
   1. When were you diagnosed with COPD?
   2. Can you tell me something about the care you receive for COPD?
   3. Which healthcare providers do you usually see for your COPD?
      1. *Prompt: General practitioner/pulmonologist/POH*
      2. Which healthcare professionals are important to you?
   4. Who else is important to you concerning your COPD care?
      1. What do they mean to you?
   5. How would you describe your role in your COPD care?
   6. What do you think of the COPD care you receive?
      1. *Prompt: Positive/negative experiences*
4. **Persons involved in COPD care process and remote patient monitoring (7 min)**

*Script: I understand that you have experience with remote patient monitoring of COPD. (Give a definition if it is not yet entirely clear what remote patient monitoring is).*

- 1. When did you start remote patient monitoring?
  2. Can you tell me more about what remote patient monitoring for COPD looks like in your care process?
     1. Could you describe what your role in remote patient monitoring?
        1. *Prompt: How do you feel about this/would you like to change something?*
  3. Which healthcare professionals are important to you in the remote patient monitoring process?
     1. Could you describe what their role?
     2. Why are they important?
  4. Who else is important to you in COPD care and the use of remote patient monitoring?
     1. Could you describe their role?
     2. Why are they important?

1. **The care process prior to a exacerbation (10 min)**
   1. Have you ever had an exacerbations?
      1. Could you describe what an exacerbation looks like for you?
   2. How do your days prior to an exacerbation look like?
      1. Do you feel it when an exacerbations seems to be initiating, if so, how?
      2. How do you recognize this?
      3. *Prompt: How does this make you feel?*
      4. What do you do if you feel an exacerbation approaching?
         1. *Prompt: Medical steps/contact with healthcare provider*

***If the patient has experience with remote patient monitoring for a long time and has already used it prior to a exacerbation****:*

- 1. What is the role of remote patient monitoring before an exacerbation occurs?
     1. *Prompt: How did you use the remote patient monitoring in the time before a exacerbation?*
        1. *How often/when?*
     2. *Prompt: Which care workers have you been in contact with at these times?*
        1. *When do you speak to them?*
        2. *How is the division between care in the hospital and care at the GP?*
        3. *What do you think of the healthcare and the care providers at these moments?*
     3. *Prompt: Who else is important and why?*
     4. How do you experience the remote patient monitoring in the period of symptom worsening?

***If the patient has not been doing remote patient monitoring for very long and has not had any experience with this prior to a exacerbation:***

- 1. How can remote patient monitoring be used to monitor your symptoms or to prevent you from having a exacerbation?
     1. *Prompt: Which healthcare workers would you like to have contact with?*
        1. *And when?*
     2. *Prompt: Who else is important?*

***Again for both patients who do and do not have experience with remote patient monitoring prior to a exacerbation:***

- 1. Do you know what to do if you think you are going to have an exacerbation or have a exacerbation?
     1. How do you know this?
     2. *Prompt: Who discussed this with you and how?*
        1. *Prompt: Could you explain what to do in this situation?*
        2. What can you do to make sure you don't have a exacerbation or have to go to the hospital?
           1. *Possibly prompt if people don't tell about remote patient monitoring: and what should you do in terms of remote patient monitoring?*

**BREAK 5 MIN**

1. **The care process during and after a exacerbation (15 min)**

***During***

- 1. What does your care look like when you have a exacerbation?
     1. How do you experience the remote patient monitoring during an exacerbation?
     2. Which healthcare professionals do you have contact with during an exacerbation?
        1. *Prompt: General practitioner or pulmonologist and nurse?*
        2. When do you speak to these people?
  2. How do you feel when you have an exacerbation?
     1. *Prompt: How do you feel mentally? (prompts: anxious, controlled, habituation, lonely/social support?)*
     2. *Prompt: How do you feel physically?*
  3. Have you ever been admitted to hospital for a exacerbation?

***If a patient has never been admitted in the event of a exacerbation:***

- 1. How do you feel about being at home during your treatment for the exacerbation?
  2. Which healthcare professional do you contact when you receive treatment for an exacerbation?
     1. What do they tell you?
     2. What information do you receive about the care after the exacerbation?
        1. Have you ever received information about remote patient monitoring after an exacerbation?
           1. If so, what kind of information do you receive?

***After recovery (only for people who have not been hospitalized)***

- 1. How do you feel when you no longer have an exacerbation?
     1. *Prompt: How do you feel* emotionally? *(prompts: anxious, controlled, habituation, lonely/social support?)*
     2. *Prompt: How do you feel physically?*
     3. What role do you think remote patient monitoring can play in your COPD care when you feel better again?
        1. *Prompt: Positive (e.g., reassurance) & negative (e.g., stirs up fear)*
  2. How is your care after an exacerbation?

***If a patient is admitted in case of a exacerbation:***

- 1. How do you feel about being hospitalized during your treatment for an exacerbation?
  2. Which healthcare professionals do you communicate with when you are hospitalized for an exacerbation?
     1. What information do you receive about the moment you can go home?
        1. *What do you think of this information (Manageable, Useful?)con*
     2. What information do you receive about remote patient monitoring when you return home?
     3. After this information, do you feel confident and certain enough to use the remote patient monitoring at home? *(Prompt: clear, complete?)*

1. ***After discharge (only for people who have been admitted)***
   1. How does the care process look like once you’re home?
      1. Which healthcare professionals do you communicate with when you return home?
      2. And how do you experience the use of remote patient monitoring during this period?
         1. When do you speak to these people?
            1. And how?
      3. What do you do when you start to feel worse again?
   2. How do you feel when you get back home?
      1. *Prompt: How do you feel* emotionally? *(prompts: anxious, controlled, habituation, lonely/social support?)*
      2. *Prompt: How do you feel physically*?
      3. How would you describe the role of remote patient monitoring during the period you start feeling better again?
         1. *Prompt: positive (e.g., reassurance) & negative (e.g., stirs up fear)*
2. **Care process remote patient monitoring general (10 min)**
   1. What do you think of remote patient monitoring care at the moment?
      1. *Prompts: Positive, Negative , Difficult/easy?*
      2. *Prompt: Possible changes? If so, what would you like to change about it now? What changes can help make it even better?*
      3. Do you think that remote patient monitoring is suitable for all people with COPD?
         1. Why or why not
      4. *Prompt: For which people with COPD is remote patient monitoring most and least suitable?*
   2. If you look at the care before and after the home measurements, what do you perceive as the biggest difference?
   3. What is important for you to use remote patient monitoring properly and why?
      1. *Prompt: Is this currently covered in remote patient monitoring?*
   4. Do you also have other chronic diseases and conditions?
      1. If so, has this changed your COPD care?
         1. *Prompt: How?*
      2. *Prompt: Do you anticipate that your chronic disease has an influence on the use of remote patient monitoring?*
         1. *How?*
   5. What do you consider as important when new remote patient monitoring products and process are developed?
      1. *Prompt: Process specific*
      2. *Prompt: Device/product specific*
3. **Closing (5 min)**
   1. Would you like to share something else about your COPD care and remote patient monitoring?
   2. May we approach you for follow-up research?
   3. Thank you for participation + VVV voucher
