# Supplementary material for: Patients’ and Health Care Professionals’ Perspectives on Remote Patient Monitoring in Chronic Obstructive Pulmonary Disease Exacerbation Management: Initiating Cocreation
Source: J Med Internet Res. 2025 May 26;27:e67666. doi: 10.2196/67666 (PMC12149775; doi:10.2196/67666)
Supplement: Multimedia Appendix 3 [file jmir_v27i1e67666_app3.docx]

### Appendix 3: Interview guide for COPD patients without experience with remote patient monitoring (approximately 60 min)

1. **Introduction (5 min)**
   1. Welcome
   2. Introduction to research and interview
   3. Consent forms
   4. Fill in the socio-demographic questionnaire
2. **Personal introduction (3 min)**
   1. Interviewer introduction
   2. Interviewee introduction
      1. Can you tell us a bit about yourself?
3. **General introduction to COPD care (5 min)**
   1. When were you diagnosed with COPD?
   2. Can you tell me something about the care you receive for COPD?
   3. Which healthcare providers do you usually see for your COPD?
      1. *Prompt: General practitioner/pulmonologist/POH*
      2. Which healthcare professionals are important to you?
   4. Who else is important to you concerning your COPD care?
      1. What do they mean to you?
   5. How would you describe your role in your COPD care?
   6. What do you think of the COPD care you receive?
      1. *Prompt: Positive/negative experiences*
4. **Introduction to remote patient monitoring (7 min)
   *You have indicated that you have no experience with remote patient monitoring/home measurements.***
   1. Have you ever heard of remote patient monitoring or home measurements? (possibly for other conditions)
   2. Have you ever heard of remote patient monitoring for patients with COPD?
      1. If so, how would you describe remote patient monitoring for COPD patients?
      2. If not, what are your expectations for remote patient monitoring in COPD care?

*NB: Should the participant not feel comfortable outlining ideas, the interviewer will give a brief and general meaning of remote patient monitoring. Then we will confirm whether this was a clear description and whether the participant has an approximate idea of the concept remote patient monitoring.*

*NB: Suppose you would have the option to use remote patient monitoring.*

- 1. What do you think you have to do yourself for remote patient monitoring?
  2. Who else do you think should be part of this?
     1. Which healthcare professional?
        1. And when?
     2. What other people?
        1. And when?

1. **The care process prior to a exacerbation (10 min)**
   1. Have you ever had an exacerbations?
      1. Could you describe what an exacerbation looks like for you?
   2. How do your days prior to an exacerbation look like?
      1. Do you feel it when an exacerbations seems to be initiating, if so, how?
      2. How do you recognize this?
      3. *Prompt: How does this make you feel?*
      4. What do you do if you feel an exacerbation approaching?
         1. *Prompt: Medical steps/contact with healthcare provider*
         2. *Prompt: Measuring with your own instruments/calling a doctor/discussing or naming complaints with those around you?*
      5. How do you experience your COPD care before you feel worse?
         1. *Prompt: Positive, negatieve?*
         2. *Prompt: What does the care look like/What kind of care do you get?*
      6. Which healthcare professionals do you communicate with within your COPD care?
         1. *Prompt: General practitioner/pulmonologist/nurse*
         2. *Prompt: When do you have contact with them?*
            1. *What is discussed?*
      7. Besides healthcare professionals, who else is important in your care when you start to feel worse?
   3. Do you know what to do if you think you are going to have an exacerbation or have an exacerbation?
      1. How do you know this?
      2. *Prompt: Who discussed this with you and how?*
         1. *Prompt: What should you do then?*
         2. What can you do to make sure you don't have an exacerbation or have to be hospitalized?
   4. How do you anticipate remote patient monitoring to play a role in this period when your symptoms worsen?

**BREAK 5 MIN**

1. **The care process during and after a exacerbation (15 min)**

***During***

- 1. What does your care look like when you have a exacerbation?
     1. How do you experience the remote patient monitoring during an exacerbation?
     2. Which healthcare professionals do you have contact with during an exacerbation?
        1. *Prompt: General practitioner or pulmonologist and nurse?*
        2. When do you speak to these people?
  2. How do you feel when you have an exacerbation?
     1. *Prompt: How do you feel mentally? (prompts: anxious, controlled, habituation, lonely/social support?)*
     2. *Prompt: How do you feel physically?*

Have you ever been admitted to hospital for a exacerbation?

***If a patient has never been admitted in the event of a exacerbation:***

- 1. How do you feel about being at home during your treatment for the exacerbation?
  2. Which healthcare professional do you contact when you receive treatment for an exacerbation?
     1. What do they tell you?
     2. What information do you receive about the care after the exacerbation?
        1. Have you ever received information about remote patient monitoring after an exacerbation?
           1. If so, what kind of information do you receive?

***After recovery (only for people who have not been hospitalized)***

- 1. How do you feel when you no longer have an exacerbation?
     1. *Prompt: How do you feel* emotionally? *(prompts: anxious, controlled, habituation, lonely/social support?)*
     2. *Prompt: How do you feel physically?*
     3. What role do you think remote patient monitoring can play in your COPD care when you feel better again?
        1. *Prompt: Positive (e.g., reassurance) & negative (e.g., stirs up fear)*

How is your care after an exacerbation?

***If a patient is admitted in case of a exacerbation:***

- 1. How do you feel about being hospitalized during your treatment for an exacerbation?
  2. Which healthcare professionals do you communicate with when you are hospitalized for an exacerbation?
     1. What information do you receive about the moment you can go home?
        1. *What do you think of this information (Manageable, Useful?)*
  3. Has the option of remote patient monitoring ever been discussed with you?
     1. If so, what has been discussed?
     2. If no, would you like to try using remote patient monitoring?
        1. *Prompt: Why or why not?*
     3. What information do you need to feel confident when start remote patient monitoring?

***After discharge (only for people who have been admitted)***

- 1. How does the care process look like once you’re home?
     1. Which healthcare professionals do you communicate with when you return home?
     2. And how do you experience the use of remote patient monitoring during this period?
        1. When do you speak to these people?
           1. And how?
     3. What do you do when you start to feel worse again?
  2. How do you feel when you get back home?
     1. *Prompt: How do you feel* emotionally? *(prompts: anxious, controlled, habituation, lonely/social support?)*
     2. *Prompt: How do you feel physically*?
     3. How would you describe the role of remote patient monitoring during the period you start feeling better again?
        1. *Prompt: positive (e.g., reassurance) & negative (e.g., stirs up fear)*

1. **Care process remote patient monitoring (10 min)**
   1. Now that we've talked a little more about remote patient monitoring, what do you think about the idea of remote patient monitoring?
      1. *Prompt: What do you like about the idea of remote patient monitoring?*
      2. *Prompt: What do you think is a less pleasant idea of remote patient monitoring?*
      3. Do you think that remote patient monitoring is suitable for all people with COPD?
         1. Why or why not
         2. *Prompt: For which people with COPD is remote patient monitoring most and least suitable?*
   2. If the option of remote patient monitoring were there, would you want to use it?
      1. *Why?*
   3. What is important for you to use remote patient monitoring properly and why?
   4. Do you have other chronic diseases and conditions?
      1. If so, has this changed your COPD care?
         1. *How?*
      2. *Prompt: Do you think these other conditions could affect remote patient monitoring?*
         1. *How?*
   5. What do you consider as important when new remote patient monitoring products and process are developed?
      1. *Prompt: Process specific*
      2. *Prompt: Device/product specific*
2. **Closing (5 min)**
   1. Is there anything else you would like to share about your COPD care and your expectations of remote patient monitoring?
   2. May we approach you for follow-up research?
   3. Thank you for participation + VVV voucher
