# Supplementary material for: Patients’ and Health Care Professionals’ Perspectives on Remote Patient Monitoring in Chronic Obstructive Pulmonary Disease Exacerbation Management: Initiating Cocreation
Source: J Med Internet Res. 2025 May 26;27:e67666. doi: 10.2196/67666 (PMC12149775; doi:10.2196/67666)
Supplement: Multimedia Appendix 4 [file jmir_v27i1e67666_app4.docx]

**Appendix 4: Themes and codes overview**

| **Themes** | **Codes** | **Code** |
| --- | --- | --- |
| Experience and care regarding COPD and exacerbations  *Description: All experiences regarding mainly exacerbations, this includes information, tips, guidance given on recognizing and anticipating lung attacks. In some cases, it also focuses on the general care obtained in COPD* | 6 | Treatment  Recognition  Medical steps  Mental and physical expression of exacerbations  Tips and guidance  Explanation/information provision |
| Rehabilitation care (secondary care)*  *Description: This mainly includes rehabilitation care and other paramedics involved, such as physiotherapist and possibly dietician. In addition, a number of codes from other themes have also been placed under this theme because the code also applied in this theme, such as tips, guidance and information given about recognizing exacerbations during the rehabilitation process. These codes are therefore often paired together.* | 10 | Requirements and wishes (added value)  Involved paramedics  Involvement/influence of patient within their own care  Patient population within rehabilitation  The rehabilitation process  The standard care process  Home care/support  Tips and guidance  Explanation/information provision  Distribution of different care processes such as primary and secondary care |
| Life with COPD*  *Description: All information given by the patient or healthcare provider that relates to living with COPD. Such as physical and mental expression or the impact on daily life. This theme and codes are mainly about the overall COPD experience and not directly focused on lung attacks. The code theme experiences regarding lung attacks is specifically focused on this.* | 11 | Co-morbidity  Daily impact  Diagnosis  Physical expression  Aids for COPD  Quality of life  Medication use  Psychological expression  Standard care process  Course of illness  COPD from the point of view of the caregiver |
| Participant characteristics and their interests  *Description: Includes all information about the participants in terms of introduction, role, and personal characteristics. All participants are placed under this theme. Also, the needs and values of participants in general within the COPD care or life are encompassed with the code "needs and values". This code was often double coded with the code "necessities and wishes" within the home monitoring theme.* | 8 | Needs and values  Introduction patient/informal caregiver  Introduction of care provider/policy officer  Knowledge of COPD  Personal characteristics  Role of care staff/policy officer  Home situation  Care needed |
| Primary care  *Description: Contacts and standard care process within primary care. However, the code "standard care process" has also been included within themes, rehabilitation care and secondary care. The code "involvement/influence of patient on care" and "distribution of different care lines" is also coded in the themes mentioned earlier.* | 7 | Involvement/influence of the patient on care  Contact GP  Contact nurse practitioner  Patient population primary care  Standard care process  Satisfaction in primary care  Distribution of different care processes such as primary and secondary care |
| Remote patient monitoring**  *Description: This theme includes all information about experiences and/or expectations in home monitoring. This also includes the wishes that participants would have if they were to apply home monitoring or the improvements they would like to see in their current use of remote patient monitoring.* | 16 | Needs and values  Requirements and wishes (added value)  Paramedics involved  Involvement of (healthcare) professionals  Contact with caregivers  Experiences  Influence of co-morbidity  Medical process/steps  Not familiar with home monitoring  Person-specific  Practical use/process-related  Product needs/requirements  Role of patient  Role of friends and family  Expectations  Concerns/critical aspects |
| Secondary care  *Description: Contacts and standard care process within secondary care. However, the code "standard care process" has also been used in the themes rehabilitation care and primary care. The code "involvement/influence of patient on care" and "distribution of different care lines" is also categorized in the other care lines.* | 8 | Paramedics involved  Involvement/influence of patient on care  Contact with pulmonologist  Contact with nurse  Patient population secondary care  Standard care process  Satisfaction of secondary care  Distribution of different care processes such as primary and secondary care |
| Care during and after an exacerbation  *Description: This theme mainly covered by the other themes, mainly by the theme,* Experience and care regarding COPD and exacerbations. *In principle, this theme includes the care that is provided during or after an exacerbation and the contact that a patient has with a healthcare provider or a healthcare institution.* | 4 | Treatment  Contact with care/care process  Tips and guidance  Explanation/information provision |

* This theme has primarily been coded to provide contextual background for the researchers and is not allocated a substantial section in the current paper.

** The codes associated with this theme are mainly relevant to the second phase of co-creation, specifically value specification, and will be explored in greater depth in a forthcoming publication.

***Note:*** *Certain codes are applicable to multiple themes and have therefore been duplicated accordingly.*
